# Perspectives on the Use of Echinocandins in the Neonatal Intensive Care Unit

**DOI:** 10.3390/antibiotics13121209

**Published:** 2024-12-12

**Authors:** Niki Dermitzaki, Foteini Balomenou, Dimitra Gialamprinou, Vasileios Giapros, Dimitrios Rallis, Maria Baltogianni

**Affiliations:** 1 Neonatal Intensive Care Unit, School of Medicine, University of Ioannina, 45500 Ioannina, Greece; n.dermitzaki@uoi.gr (N.D.); f.balomenou@uoi.gr (F.B.); drallis@uoi.gr (D.R.); mbalt@doctors.org.uk (M.B.); 2 Second Neonatal Department and Neonatal Intensive Care Unit (NICU), “Papageorgiou” University Hospital, Aristotle University of Thessaloniki, 56403 Thessaloniki, Greece; gialamprinou@gmail.com

**Keywords:** neonatal candidiasis, invasive *Candida* infections, antifungal treatment, echinocandins, micafungin, caspofungin, anidulafungin

## Abstract

The neonatal intensive care unit (NICU) population, especially low birth weight and critically ill neonates, is at risk of invasive *Candida* infections, which are associated with high mortality rates and unfavorable long-term outcomes. The timely initiation of an appropriate antifungal treatment has been demonstrated to enhance the prognosis. Factors that should be considered in the choice of an antifungal agent include the causative *Candida* strain, the presence and location of deep tissue infection, any previous use of antifungal prophylaxis, and the presence of implanted devices. Amphotericin B and fluconazole, the first-line drugs for neonatal candidiasis, are not always suitable due to several limitations in terms of efficacy and adverse effects. Therefore, alternative antifungals have been studied and used in neonates when conventional antifungals are ineffective or contraindicated. This narrative review aims to provide an overview of the current literature regarding the use of echinocandins in the neonatal population. The three echinocandins, micafungin, caspofungin, and anidulafungin, share characteristics that make them useful for the treatment of neonatal candidiasis, including activity against a wide range of *Candida* strains and *Candida* biofilms and a favorable safety profile.

## 1. Introduction

### 1.1. Invasive Candida Infections in Neonates

*Candida* spp. comprises a genus of fungi that, while part of the normal human flora, has the potential to cause both superficial and invasive diseases in humans. Neonates represent a population at risk of systemic *Candida* infections, especially preterm and very low birth weight (VLBW) neonates, and invasive candidiasis (IC) is a common cause of late-onset neonatal infection associated with high mortality and adverse outcomes [1,2,3].

The incidence of IC is inversely related to gestational age and birth weight and is estimated to affect 4–18% of critically ill neonates in neonatal intensive care units (NICUs) [4]. Extremely low birth weight (ELBW) neonates are at significant risk of adverse outcomes, with mortality rates reaching 50% and a risk of adverse neurodevelopmental outcomes exceeding 40% [3,5].

*Candida* spp. have the potential to disseminate through the circulatory system and invade various body tissues, leading to deep-tissue infections [6]. Central nervous system (CNS) involvement is common in neonatal candidiasis due to the immature blood–brain barrier, affecting 10–20% of patients [7]. However, the incidence of hematogenous *Candida* meningoencephalitis (HCME) increases significantly in ELBW neonates and has been reported to be as high as 60% [5,8]. Given that cerebrospinal fluid (CSF) parameters and imaging may be normal despite the presence of CNS involvement and the high rates of HCME in neonates, the choice of antifungal agent in neonatal IC should be made in the presumed diagnosis of meningoencephalitis [9,10].

*Candida albicans* is the most prevalent strain in neonatal candidiasis. Among the non-*albicans* species, whose prevalence is steadily increasing, especially in middle- and low-income countries, *Candida parapsilosis* is the most commonly isolated strain, followed by *Candida glabrata, Candida tropicalis, Candida krusei*, and the emerging *Candida auris*. However, the distribution of *Candida* strains varies significantly across different geographical locations. The susceptibility profiles of different *Candida* strains vary; therefore, accurate strain identification is essential for selecting an appropriate antifungal treatment [3,11,12,13,14,15,16].

### 1.2. Diagnosis of Invasive Candida Infections in Neonates

Clinical diagnosis of IC in neonates is particularly challenging as the clinical picture is indistinguishable from that of bacterial sepsis. Moreover, the available laboratory diagnostic techniques have significant limitations. Blood culture, which is considered the gold standard for IC diagnosis, has a sensitivity of up to 50% and a slow turn-around time [17,18]. Despite the high negative predictive value of serum biomarkers, mannan/anti-mannan antibodies, and 1,3 β-D-glucan have significant disadvantages in terms of specificity, which restrict their clinical utility [19,20]. Improved sensitivity and specificity have been achieved with recently developed techniques, including polymerase chain reaction (PCR), next-generation sequencing (NGS), and T2 magnetic resonance (T2MR); however, their high cost prevents their use in most settings [21]. Precise and rapid diagnosis is the prerequisite for early and effective antifungal treatment administration, associated with improved survival and long-term morbidity outcomes.

### 1.3. Antifungal Drugs in Neonatal Candidiasis

The following four classes of antifungal agents have been studied and utilized in neonates with IC: polyenes, triazoles, nucleoside analogues, and echinocandins. Amphotericin B deoxycholate, a polyene active against various *Candida* spp., has been used for decades in the treatment of neonatal IC; however, adverse effects such as nephrotoxicity are not uncommon, although less frequent in neonates than in adults [22,23]. Fluconazole, a first-class triazole with a favorable safety profile and excellent oral bioavailability, is widely used in NICUs for the treatment of IC and as antifungal prophylaxis in high-risk neonates. However, increasing resistance among *Candida* spp. and its previous use as a prophylactic agent often preclude its use [24,25]. Flucytosine, a nucleoside analogue, is only used in combination with other antifungals because of the rapid emergence of resistance. Due to its excellent CNS penetration, it is often used as adjunctive therapy in combination with amphotericin B for *Candida* meningitis [26,27]. The echinocandins micafungin, caspofungin, and anidulafungin are more recently developed and less studied antifungals compared to the previously described categories, and they are more commonly used as salvage therapy in neonatal IC. Among the echinocandins, micafungin is the only one approved for use in neonates.

The current Infectious Diseases of North America (IDSA) and European Society of Clinical Microbiology and Infectious Diseases (ESCMID) guidelines, published in 2016 and 2012, respectively, recommend amphotericin B deoxycholate (D-Amb) or fluconazole as the first-line treatment for neonatal IC. However, fluconazole is only recommended for neonates who have not previously received prophylaxis with this agent. Flucytosine is suggested as salvage therapy in neonates with CNS candidiasis who are unresponsive to amphotericin monotherapy. Both authorities suggest lipid formulations of amphotericin or the echinocandins micafungin and caspofungin as alternatives to D-Amb and fluconazole. In particular, echinocandins are recommended in cases where the use of first-line drugs is precluded by resistance or toxicity [10,28].

Although extensively studied in neonates, conventional antifungals, D-Amb and fluconazole, are sometimes inappropriate or contraindicated. D-Amb is generally considered to be a well-tolerated agent, but it targets ergosterol in the fungal cell membrane, which is also present in the mammalian cell wall, and this can lead to adverse effects, including nephrotoxicity and hepatotoxicity [23,29]. As fluconazole prophylaxis in high-risk neonates is a widely implemented strategy in NICUs, fluconazole-resistant *Candida* spp. tends to emerge. The potential utility of an antifungal agent active against fluconazole-resistant *Candida* spp. is indisputable [24,30]. In addition, conventional antifungals have a limited efficacy against a number of non-*albicans Candida* spp., including *Candida auris*, as well as against *Candida* biofilms [31,32]. Echinocandins represent a class of antifungal agents that may be appropriate for use in neonates in cases where conventional antifungal agents have been demonstrated to be ineffective.

The objective of this narrative review is to present an overview of the current literature on the clinical use of echinocandins in NICUs.

## 2. Materials and Methods

The PubMed and Google Scholar databases were scanned for relevant studies up to October 2024 with the following keywords: neonatal invasive candidiasis, preterm neonate, candidiasis treatment, antifungal agents, echinocandins, micafungin, caspofungin, and anidulafungin. Randomized control trials (RCTs), systematic reviews, narrative reviews, observational studies, and case reports were included. In addition, the reference lists of the retrieved articles were screened to assess the presence of relevant studies that might not have been identified in the initial search. The methodology employed in the present study for the identification of the relevant literature is outlined in Table 1.

The current narrative review is structured as follows:

(1) The echinocandin class of antifungals—basic information.
(2) Echinocandins in the neonatal population—pharmacokinetics and dosing, efficacy, and adverse effects.
  (i) Micafungin.
  (ii) Caspofungin.
  (iii) Anidulafungin.

(3) Special aspects of echinocandins in NICUs.
  (i) *Candida auris* treatment.
  (ii) Activity against biofilms.

(4) Optimizing empiric antifungal use in the NICU.

## 3. Echinocandins (Mode of Action, Therapeutic Range, Safety Profile)

Echinocandins are semisynthetic lipoproteins that exert their antifungal activity by inhibiting the enzyme complex 1,3 β-D-glucan synthase. This disrupts the synthesis of 1,3-beta-D-glucan, a component of the fungal cell wall essential for its structural integrity, leading to structural deformations and cell lysis due to osmotic imbalance [4,33,34].

The glucan synthase complex consists of two main subunits, the catalytic unit FKS1p and the regulatory protein Rho1p. The FKS1p subunit, which is encoded by the FSK1, FSK2, and FSK3 genes, is responsible for regulating cell wall remodeling and serves as the binding unit for echinocandins, leading to the inhibition of synthase activity. The protein Rho1p, a GTPase, regulates 1,3-beta-D-glucan synthesis [33,35]. Echinocandin antifungal activity against different fungal species depends on the density of glucan in the cell wall, which varies between species and is associated with fungicidal activity against distinct *Candida* spp. [4,26].

Echinocandins demonstrate an in vitro and in vivo concentration-dependent activity and a prolonged post-antifungal effect against *Candida albicans* and against a wide range of non-*albicans* spp., even strains resistant to azole or amphotericin such as *C. krusei*, *C. glabrata*, and *C. lusitaniae* [32,36,37,38]. The recently emerged multidrug-resistant *C. auris* is generally susceptible to echinocandins, which are the drug of choice beyond the neonatal period [39,40]. However, members of this class are less effective against *C. parapsilosis*, probably due to the intrinsic mutations of this strain in the FKS gene [41,42]. Notably, echinocandins have been shown to be effective against *Candida* biofilms, which represent a significant therapeutic challenge, particularly in intensive care units (ICUs) [43].

The poor gastrointestinal absorption of echinocandins precludes oral administration, and all formulations are exclusively administered parenterally [35]. Echinocandins exhibit high protein binding (>99%) and broad tissue distribution, including the lungs, liver, spleen, and gastrointestinal tract. However, due to their high molecular weight and extensive binding to plasma proteins, ocular and central nervous system penetration is limited [33,35,37]. Degradation by hydrolysis and N-acetylation occurs mainly in the liver but also in the spleen and adrenal glands, and the deactivated compounds are slowly eliminated via the biliary system [35,37].

Echinocandins are generally considered to have a favorable safety profile due to the absence of their target enzyme, 1,3 β-D-glucan synthase, in mammalian cells [4,35]. Indeed, a meta-analysis reported fewer discontinuations due to adverse effects in children treated with echinocandins compared to other antifungals [44]. In addition, the potential for interaction with other drugs is minimal as echinocandins are not substrates for cytochrome p450 or the P-glycoprotein transport system [45].

Among the echinocandins, micafungin, caspofungin, and anidulafungin have been approved by the Food and Drug Administration (FDA) and the European Medicines Agency (EMA) for the treatment of IC in adult populations [46]. While all echinocandins are labeled for pediatric use, micafungin is the only one approved by the FDA and EMA for the treatment of neonatal candidiasis [47,48].

## 4. Micafungin

Micafungin, derived from *Coleophoma empetri*, is the most studied echinocandin for the treatment of neonatal candidiasis and is the only echinocandin with both EMA and FDA approval for use in the neonatal population [33,47,48].

### 4.1. Pharmacokinetics and Dosing

Pharmacokinetic and pharmacodynamic studies have revealed significant discrepancies in the pharmacokinetics of micafungin in neonates compared to older children and adults (Table 2). The clearance of micafungin has been demonstrated to be higher in neonates; however, the underlying mechanism remains unclear. It has been proposed that the lower plasma-binding protein and albumin levels in neonates result in a higher fraction of unbound drug and, therefore, faster elimination [49,50]. Yanni et al. demonstrated that the unbound fraction of micafungin in neonatal plasma was eight times higher than that in adult plasma, providing evidence in support of the hypothesis that the age-dependent serum protein binding of micafungin is responsible for the enhanced clearance [51]. The correlation between body weight and clearance is not linear [33]. Hope et al., using a two-compartment model, demonstrated that micafungin clearance is allometrically scaled to body weight and that aspartate transaminase (AST) and bilirubin represent covariates with a substantial effect. Furthermore, it was observed that in neonates and young children, the predicted clearance based solely on body weight resulted in an estimation lower than the actual clearance [52].

It has been demonstrated that micafungin clearance in neonates is approximately 0.036 L/h/kg, approximately four times higher than the expected clearance in adults [36,53,54,55]. Micafungin clearance decreases with a higher body weight and age [49]. De Rose et al., in a pooled analysis of two studies involving 53 preterm and term neonates with IC treated with micafungin, reported higher clearance and shorter half-life in neonates before 28 days (Cl: 0.036 L/h/kg, Τ1/2: 13.5 h) compared with infants after 120 days of postnatal age (Cl: 0.028 L/h/kg, Τ1/2: 14.4 h) [49]. Benjamin et al. observed a 21% higher clearance of micafungin in ELBW neonates than in neonates with a greater birth weight [55]. Additional studies have also reported increased clearance in neonates with a birth weight of less than 1000 g compared to higher birth weights [53,54].

**Table 2:** Clinical studies evaluating the pharmacokinetics of micafungin in neonates.

Author	Population	Dose	Clearance (L/h/kg)	Volume of Distribution (L/kg)	T1/2 (h)	AUC_0–24_ (mg·h L^−1^)
Hereshi, 2008 [54]	18 preterm, BW > 1000 g	0.75 mg/kg/day (ν = 6),	0.0398	0.435	8.3	19.0 (0.75 mg/kg)
		1.5 mg/kg/day (ν = 6)				34.5 (1.5 mg/kg)
		3 mg/kg/day (ν = 6)				69 (3 mg/kg)
Smith, 2009 [53]	12 preterm	15 mg/kg/day	0.0345	0.620	No data	437.5
			(0.030 BW > 1000 g, 0.037 BW < 1000 g)			(472.2 BW > 1000 g, 412.7 BW < 1000 g)
Benjamin, 2010 [55]	13 neonates		0.0300	0.450	11.0	275.2
		7 mg/kg/day (BW > 1000) 10 mg/kg/day (BW < 1000)	(0.024 BW > 1000 g, 0.036 BW < 1000 g)	(0.34 BW > 1000 g, 0.54 BW < 1000 g)	(11.4 BW > 1000 g, 10.6 BW < 1000 g)	(291.2 BW > 1000 g, 258.1 BW > 1000 g)
Benjamin, 2018 [56]	20 neonates	10 mg/kg/day	0.0292	0.480	12.2	399.3
Leroux, 2018 [57]	18 neonates	10 mg/kg/day	0.020	0.354	13.6	478.9
De Rose, 2023 [49]	53 neonates	8–15 mg/kg/day	0.036 (<28 days)	No data	13.5 (<28 days)	259.0 (<28 days)
			0.028 (>120 days)		14.4 (>120 days)	346.4 (>120 days)

It is well recognized that the body composition of neonates, with higher total body water, lower adipose tissue, and plasma proteins, significantly affects drug distribution compared to older children and adults, resulting in a greater volume distribution [34,49]. Consistently, pharmacokinetic studies of micafungin in the neonatal population have demonstrated a volume distribution in neonates of 0.34–0.62 L/kg which is significantly larger than that reported in adults (0.26 L/min) [49,53,54,55,56,57].

The fungicidal activity of micafungin is concentration-dependent [4]. Due to the significant pharmacokinetic differences between micafungin in neonates and adults and the high prevalence of CNS involvement in neonatal candidiasis, higher weight-based doses are required in the neonatal population compared to older children and adults to achieve adequate drug exposure. The recommended dose for the treatment of invasive candidiasis in adults is 100 mg/day. In neonates and infants under four months of age, it is estimated that the equivalent drug exposure is achieved with doses of micafungin of 4 mg/kg/day [48]. Moreover, it has been reported that drug exposure at a dose of 15 mg/kg/day in neonates is equivalent to a dose of 5 mg/kg/day in adults [53].

Due to its high molecular weight and high protein binding, micafungin has been shown to have poor CSF penetration in adults, which is of particular concern for the treatment of neonatal candidiasis. CNS involvement, typically HCME, is common, particularly in ELBW neonates, reported in up to 63% of cases of candidiasis [5]. However, CNS penetration has been demonstrated to be concentration-dependent, and higher doses effectively eradicate CNS *Candida* spp. [9].

A bridging study of HCME in a rabbit model indicated that micafungin doses above 9 mg/kg/day are necessary to achieve CNS concentrations capable of eradicating *Candida* spp. in cases of neonatal HCME. The highest concentrations of the drug were found in the meninges and the choroid [58]. Furthermore, a recent study showed that higher doses of micafungin resulted in higher CSF drug levels. The authors concluded that doses between 8 and 15 mg/kg/day could cross the blood–brain barrier and attain therapeutic levels of micafungin in the CSF [49]. It has been suggested that CNS penetration is probably independent of meningeal inflammation [58].

Given the low urinary excretion of active micafungin, its therapeutic utility in treating urinary tract candidiasis, a common sequela of neonatal IC, is limited [59]. However, micafungin has been used as an alternative for urinary candidiasis due to the rising prevalence of non-*albicans* species resistant to first-line antifungals. An increasing number of successful treatments of *Candida* urinary tract infections with micafungin cases are being reported [59,60,61]. It has been suggested that although urinary excretion is limited, high plasma levels provide sufficient urinary levels to achieve an optimal urinary concentration and eradicate *Candida* spp. effectively [60].

Various micafungin doses ranging from 0.75 mg/kg/day to 15 mg/kg/day have been evaluated for pharmacokinetics and their efficacy in the neonatal population. An in vitro-to-clinical bridging study in a rabbit model demonstrated that a micafungin area under the curve (AUC)_0–24_ > 166.5 mg·h L^−1^ is necessary for the treatment of neonatal candidiasis, in which HCME cannot be excluded [58]. A multicenter study of micafungin pharmacokinetics in preterm infants treated with doses of 0.75–3 mg/kg/day showed that low plasma drug concentrations were achieved [54]. Benjamin et al. reported the achievement of target AUC in both neonates >1000 g birthweight treated with micafungin 7 mg/kg/day and neonates <1000 g treated with 10 mg/kg/day [55]. According to the TINN study results, all 18 neonates treated with micafungin 10 mg/kg/day achieved the target AUC_0–24_ of 166.5 mg·h L^−1^ from the first day of treatment. The authors also demonstrated that the target AUC_0–24_ was achieved regardless of whether or not a loading dose of 15 mg/kg was administered [57]. Another population pharmacokinetic study reported that 82.6% of neonates treated with micafungin 10 mg/kg/day achieved the target AUC_0–24_ [62]. In a population of preterm neonates that received micafungin 15 mg/kg/day, Smith et al. reported a mean AUC_0–24_ of 437.5 mg·h L^−1^ and the highest AUC_0–24_ of 555.6 mg·h L^−1^ [53].

According to the EMA product license and the latest ESCMID guidelines, micafungin doses of 4–10 mg/kg are recommended for neonates and infants <4 months of age, with the higher doses being indicated for cases where CNS involvement is established or cannot be excluded [48,63]. The FDA recently approved micafungin 4 mg/kg/day for the treatment of neonatal candidiasis without CNS involvement. As indicated in the product license, the safety of higher doses required to achieve adequate drug exposure in the CNS has not been established and is therefore not recommended [47].

### 4.2. Efficacy

Several studies have investigated the efficacy of micafungin in the treatment of neonatal candidiasis. In a recent prospective study, Auriti et al. reported that 61.9% of neonates with IC treated with 8 mg/kg/day of micafungin achieved *Candida* spp. eradication. Among those who received treatment for more than 14 days, the success rate rose to 86.7% [64]. De Rosa et al. reported mycological eradication in 83.3% of the neonates with IC treated with micafungin, with higher success rates in neonates treated with higher doses (8–10 mg/kg/day). The authors demonstrated, through the analysis of the probability of target attainment (PTA), that doses of 4–8 mg/kg may be effective against *Candida* spp., with a minimum inhibitory concentration (MIC) of less than 0.064 mg/L. Conversely, species with a MIC from 0.064 to 0.125 mg/L may require higher doses of 10 mg/kg to be effective [49].

Benjamin et al. aimed to compare the efficacy of micafungin 10 mg/kg/day with D-Amb in infants <120 days with IC in a phase 3, multicenter, randomized, double-blind trial. The study was terminated after two years due to low recruitment, having enrolled 20 and 10 neonates in the micafungin and D-Amb groups, respectively. Efficacy was not significantly different between the two groups, with 61% mycological clearance after one week of treatment in the micafungin group and 70% in the D-Amb group. It should be noted that five out of seven cases in the micafungin group were classified as treatment failure due to the absence of an assessment of the mycological response. In contrast, all cases of persistent infection in the D-Amb group were confirmed. The two infants unresponsive to micafungin treatment were infected by *C. parapsilosis* [56]. *C. parapsilosis,* the most common non-*albicans Candida* spp. implicated in neonatal infections, has higher in vitro MICs to micafungin than other *Candida* spp., prompting concerns about its in vivo efficacy. However, this has not been proven, and clinical trials in adults do not favor other antifungals over echinocandins for *C. parapsilosis* infections [10,65,66,67]. Multidrug-resistant *C. auris* is generally susceptible to echinocandins. Chandramati et al., in a retrospective cohort study of neonates with *C. auris* infection, reported that the addition of micafungin to the antifungal regimen (micafungin plus amphotericin) reduced the mortality rate from 75% to 0% [68].

### 4.3. Adverse Effects

Several studies have evaluated the safety of micafungin in the neonatal population and concluded that it is a well-tolerated agent with a favorable safety profile [49,54,55,57,64,69,70]. The most commonly reported adverse events in neonates treated with micafungin are mild and transient and include liver enzyme elevation, gastrointestinal symptoms, hypokalemia, and fever [24].

In a cohort of 35 preterm neonates and infants younger than 180 days old, Auriti et al. reported that cessation of treatment or dose reduction was not necessary in any infant due to adverse events. However, transient liver enzyme elevation was observed in 20% of the study population [64]. De Rose et al. observed a transient increase in liver enzymes in 5.7% of newborns treated with micafungin 8–15 mg/kg/day, which resolved after completion of treatment [49]. However, a case of transient severe hepatitis and cholestasis has been reported in a preterm infant treated with micafungin 8 mg/kg/day [71]. A black box warning has been issued for micafungin due to potential hepatotoxicity based on the development of hepatocellular tumors in rats treated with micafungin. However, the doses administered to the experimental animals were eight times higher than those used in humans, and the treatment duration was over three months [48,49]. A systematic review of nine studies concluded that micafungin is safe for preterm and term neonates. Furthermore, the occurrence of adverse events was found to be independent of the administered dose or treatment duration [70].

Micafungin has a low potential for interactions with other pharmaceutical agents. The absence of involvement of the cytochrome P450 and the P-glycoprotein metabolic pathway diminishes the potential for interaction with other pharmaceutical agents. Moreover, it has been demonstrated that although micafungin is a substrate for CYP3A in vivo, this pathway does not contribute significantly to the drug’s metabolism. This is of significant importance in the NICU population, where co-administration with other drugs is often a necessity. In particular, concomitant administration of antifungals with antibiotics, such as aminopenicillins, aminoglycosides, cephalosporins, and carbapenems, is commonly required in critically ill neonates. In consideration of its pharmacokinetic profile, micafungin represents a suitable option, and no dosage modifications are necessary when co-administered with antibiotics commonly used in neonates [48,72,73,74].

Nevertheless, there is a low possibility of drug–drug interaction with agents that are metabolized through the CYP3A pathway. The concomitant administration of micafungin and D-Amb has been observed to result in a 30% increase in plasma levels of the latter, increasing the potential for side effects of D-Amb [48,73].

## 5. Caspofungin

Caspofungin is a cyclic semisynthetic lipopeptide derived from Pneumocandin B_0_, a lipohexapeptide produced by the yeast *Glarea lozoyensis*. It was the first echinocandin to be approved by the FDA for the treatment of IC in adults in 2001 and later for pediatric patients over three months of age in 2008 [35]. The FDA and EMA do not currently approve the use of caspofungin in neonates.

### 5.1. Pharmacokinetics and Dosing

Similarly to the other echinocandins, caspofungin shows high protein binding and wide tissue distribution [33]. Caspofungin exhibits modest non-linear pharmacokinetics with the potential for drug accumulation at higher doses [74,75]. After caspofungin infusion, there is a short alpha-phase, followed by a beta-phase with a half-life of 10–15 h, which has a log-linearity resulting in a 10-fold decrease in drug concentration, and a prolonged gamma-phase with a half-life of 40–50 h, which is responsible for the accumulation of caspofungin [74]. It has been demonstrated that the rate of decline in plasma concentration during the beta phase is faster in children than in adults. The higher clearance and the decreased volume of distribution of the drug in the pediatric population is probably the explanation for the reduced half-life of the beta-phase compared to adults [76,77].

According to pharmacokinetic studies, the clearance of caspofungin is lower in neonates, increases during childhood, and then progressively decreases during adolescence and adulthood [78]. The main process affecting plasma clearance is the distribution of the drug rather than its elimination [79]. It has been demonstrated that the distribution of caspofungin to tissues is achieved via uptake transporters; more specifically, the OATP1B1 transporter mediates the hepatic uptake. The developmental immaturity of neonates, associated with reduced transporter expression, may be responsible for the reduced hepatic distribution and clearance observed in the neonatal population, along with physiological factors such as changes in blood flow rates and organ mass [78,80]. The lower caspofungin clearance, the larger volume of distribution, and the low level of plasma proteins in neonates, particularly preterm neonates, potentially expose them to the risk of drug accumulation [81].

Caspofungin is metabolized in the liver by spontaneous degradation to an open-ring peptide and by hydrolysis and N-acetylation to deactivated products [33,35,75]. Elimination is slow, and metabolites are mainly excreted in feces and urine. Only a minimal proportion of caspofungin, about 1.4%, is excreted unchanged in the urine [74].

Data on the pharmacokinetics of caspofungin in neonates are sparse, and the suggested doses are mostly extrapolated from studies on older children and adults. Caspofungin exhibits a concentration-dependent activity against *Candida* spp. [75]. Several pharmacokinetic studies have shown that dosing based on body surface area (BSA) is the preferred approach in infants and children [82].

Walsh et al. conducted the first prospective study of the pharmacokinetics of caspofungin in children and adolescents, with the aim to compare the weight-based dose of 1 mg/kg/day and the BSA-based dose of 50 mg/m^2^/day or 70 mg/m^2^/day with data from pharmacokinetic studies in adults treated at the recommended dose of 50–70 mg/day. They concluded that the weight-based dose of 1 mg/kg/day results in suboptimal caspofungin concentrations and that the BSA-based dose of 50 mg/m^2^/day achieves a drug exposure equivalent to that of adults receiving 50 mg/day. Although the measured AUC_0–24_ of caspofungin on the first day of administration was higher in children than in adults, the AUC_0–24_ at a steady state was comparable, and the initial increased drug exposure was considered clinically insignificant [76]. Similar conclusions were reached in a subsequent study of infants and toddlers aged 10–23 months who received caspofungin 50 mg/m^2^/day [77]. Another prospective pharmacokinetic study in children aged 2–12 years using the aforementioned dosing regimen showed that more than 95% of children achieved concentrations above the target MIC of 1 μg/L [82]. A number of studies have shown that in children aged 3 months to 17 years, a caspofungin loading dose of 70 mg/m^2^ followed by a daily dose of 50 mg/m^2^ is associated with drug exposures within the target range and is the currently recommended dosing regimen for this age group [10,28,83,84,85,86].

The existing literature on the pharmacokinetics of caspofungin in neonates is limited. In a prospective multicenter study, Saez-Llorens et al. evaluated the plasma levels and tolerability of caspofungin at a dose of 25 mg/m^2^/day in neonates and infants less than three months of age. Drug exposure was found to be comparable to that of adults receiving 50 mg/day; however, a slightly increased trough concentration was observed in neonates. In comparison to the concentration levels observed in children and adolescents who received 50 mg/m^2^/day, the peak concentration of caspofungin was found to be reduced, while the trough concentration was increased in neonates who received 25 mg/m^2^. This is consistent with the data indicating that caspofungin clearance is lower in neonates and increases during childhood [78].

In a recent multicenter RCT involving neonates treated with caspofungin 2 mg/kg/day, serum concentrations on days 4 and 7 of the treatment were compared, and no significant difference was found. Therefore, the authors concluded that a steady state was attained by the fourth day of treatment [87].

Jans et al. described the successful treatment of shunt-associated *Candida* meningitis in a preterm neonate with caspofungin 25 mg/m^2^/day and studied drug concentrations in plasma and CSF with serial sampling. The results demonstrated an adequate CSF penetration and a potentially beneficial reduced clearance of caspofungin in the CSF in comparison to serum. Several factors were recognized as potential contributors to the successful *Candida* spp. CSF eradication, including the immaturity of the neonatal blood–brain barrier, the high systemic caspofungin exposure associated with higher CSF drug concentrations, and the low protein content of CSF resulting in the increase in unbound and therefore active drug [88].

### 5.2. Efficacy

Data on the efficacy of caspofungin in the treatment of IC in the neonatal population are limited, as only a small number of trials and case reports have been published (Table 3).

Caspofungin is often considered a salvage therapy in patients with candidemia refractory to conventional antifungals, and several cases of the successful eradication of *Candida* spp. after the addition of caspofungin in preterm infants with persistently positive cultures, despite prolonged antifungal administration, have been reported [28,82,89,90,91,92]. Natarajan et al. reported successful eradication in 11 out of 13 neonates with IC refractory to conventional therapy after a median treatment duration of 14.5 days, including two cases of meningitis, following the addition of caspofungin 1 mg/kg/day to the treatment regimen [93]. Furthermore, Jeon et al. reported the successful eradication of *Candida* spp. in seven ELBW with persistent candidiasis after 6–36 days of amphotericin after administering 3–13 days of caspofungin 2 mg/kg/day [94]. Odio et al. reported the eradication of *Candida* spp. within 4.3 days following caspofungin monotherapy in ten neonates previously unsuccessfully treated with amphotericin [95]. A case of successful eradication of shunt-associated *C. albicans* meningitis refractory to a 4-week treatment with fluconazole and amphotericin in a preterm neonate three days after caspofungin 25 mg/m^2^ was added to the regimen without the removal of the medical device [88].

Mohamad et al. compared the efficacy of caspofungin 2 mg/kg/day with amphotericin 1 mg/kg/day in neonates with confirmed IC in a double-blind RCT. Effective treatment was defined as the resolution of symptoms and mycological eradication confirmed by negative culture, in addition to the absence of breakthrough fungal infection and survival for at least 7 days after the cessation of treatment. Caspofungin was demonstrated to be significantly more effective than amphotericin (86.7% vs. 41.7%, respectively). Notably, the majority of neonates were diagnosed with deep tissue infections, including meningitis, endophthalmitis, and urinary tract infections, which are considered to be sites where echinocandins have a relatively limited penetration [96]. A recent multicenter, double-blind, phase 2 RCT also compared the efficacy of caspofungin to that of amphotericin B, defined as mycological eradication two weeks after treatment completion in infants younger than three months with systemic candidiasis. No significant difference was observed regarding efficacy between the two antifungal agents, with a success rate of 71% for caspofungin and 68.8% for amphotericin [87].

**Table 3:** Clinical studies on the safety and efficacy of caspofungin in neonates.

Author	Population	Candida Isolates	Dose	Microbiologic Cure	Drug-Related Adverse Effects
Odio, 2005 [95]	11 neonates (9 preterm, 1 term) with candidemia refractory to conventional antifungals	*C. albicans* (4) *C. parapsilosis* (3) *C. tropicalis* (2) *C. glabrata* (1)	1 mg/kg/day for 2 days, followed by 2 mg/kg/day Monotherapy	11/11 (100%)	No
Natarajan, 2005 [93]	13 neonates (12 preterm, 1 term) with candidemia refractory to conventional antifungals	*C. albicans* (5) *C. parapsilosis* (6) *C. albicans*/C. *parapsilosis* (1) *C. tropicalis* (1)	1 mg/kg/day (5/13 loading dose of 1.5 mg/kg/day) Co-administered with conventional antifungals	11/13 (84.6%)	Thrombophlebitis (1) Hypokalemia (2) Elevated liver enzymes (4) Direct hyperbilirubinemia (1)
Mohamed, 2012 [96]	15 neonates	*C. albicans* (11) *C. parapsilosis* (3) *C. tropicalis* (1)	2 mg/kg/day Monotherapy	13/15 (86.7%)	Hypokalemia (2) Elevated liver enzymes (1) Hyperbilirubinemia (1) Elevated creatine (1) Thrombophlebitis (1) Rash (1) Fever (1) Vomiting (1)
Jeon, 2013 [94]	7 ELBW neonates with candidemia refractory to conventional antifungals	*C. albicans* (1) *C. parapsilosis* (6)	2 mg/kg/day	6/7 (85.7%)	No
Kim, 2020 [87]	31 infants <3 months	*C. albicans* (27) *C. parapsilosis* (1) *C. tropicalis* (1) *C.glabrata/C. albicans* (1) *C. intermedia* (1)	2 mg/kg/day Monotherapy	22/31 (71%)	Infusion site edema (1) Cholestatic jaundice (1)

### 5.3. Adverse Effects

Similarly to the other echinocandins, caspofungin has been demonstrated to be well tolerated in pediatric patients. The most common adverse effects related to therapy are mild and usually do not necessitate treatment discontinuation. These include fever, rash, transient elevation of liver enzymes, and hypokalemia [97].

Despite the limited number of prospective studies that have investigated the safety of caspofungin in the neonatal population, the available data, including those from clinical cases, suggest that it has a favorable safety profile in neonates (Table 3). It is noteworthy that the administration of caspofungin did not result in any adverse events in cohorts of preterm neonates [94,95]. Moreover, Natarajan et al. reported no significant adverse effects when caspofungin was administered concurrently with other antifungal agents. The transient elevation of liver enzymes was the most common side effect, occurring in four out of the twelve neonates [93].

A recent RCT comparing caspofungin with amphotericin in neonates with IC demonstrated that neonates in the caspofungin group experienced fewer adverse events than those in the amphotericin group. Among the thirty-four neonates receiving caspofungin at a dose of 2 mg/kg/day, two patients developed drug-related adverse events, one cholestatic jaundice, and one edema at the injection site. However, there were no adverse events requiring the discontinuation of the treatment [87].

## 6. Anidulafungin

Anidulafungin is the most recently developed echinocandin derived by modulating the chemical structure of echinocandin B, and it is the first echinocandin discovered [35]. Despite being licensed for the treatment of IC in adults since 2006, anidulafungin has only been approved by the FDA for use in pediatric patients beyond the first month of life in 2020 [98,99].

### 6.1. Pharmacokinetics and Dosing

Anidulafungin exhibits similar characteristics to other echinocandins, displaying high protein binding and a wide distribution across tissues. In autopsy tissues, Marx et al. demonstrated that anidulafungin concentrations in various tissues, including the liver, lung, and kidneys, achieved therapeutically effective levels [100]. The same group of authors examined anidulafungin concentrations in CSF and brain tissue and concluded that these were below the MIC for a number of *Candida* species [101]. However, Ripp et al. showed that in experimental neonatal rats, the ratio of anidulafungin concentration in brain tissue to plasma was about 0.2 after the first dose but increased to 0.7 after the fifth dose [102].

Clearance of anidulafungin is achieved by slow non-enzymatic degradation in the blood and its biotransformation into an open-ring inactive compound. The breakdown products are eliminated mainly through bile excretion and elimination in the feces [98]. Given that the liver is not involved in drug degradation, and biliary excretion represents the primary route of elimination, dose adjustment is not necessary in patients with impaired hepatic or renal function [35].

The available evidence from studies of adults and the very limited data from studies of neonatal and pediatric populations indicate that anidulafungin exhibits linear and predictable pharmacokinetics across these different populations [103] (Table 4). In contrast to micafungin and caspofungin, the clearance of anidulafungin has been shown to be independent of age [33,103,104]. Based on pharmacokinetic studies’ findings, anidulafungin clearance shows a linear relationship with body weight, from approximately 0.015 L/kg/h in neonates to 1 L/h in adults [103]. Cohen-Wolkowiez et al. conducted the only pharmacokinetic study of anidulafungin in neonates and found that the half-life of anidulafungin decreased with an increase in age. However, the difference between neonates and infants was not statistically significant. Furthermore, the authors demonstrated that a steady state is probably reached after the loading dose, as no drug accumulation was observed after multiple doses of anidulafungin [105]. A pooled population analysis demonstrated that the systemic exposure of anidulafungin was comparable across different age groups, from neonates to adults, following the administration of a weight-based dose in pediatric patients (3 mg/kg loading dose and 1.5 mg/kg/day thereafter) and the standard adult dose (200 mg loading dose and 100 mg/day thereafter), with an estimated AUC of 80.77 mg·h L^−1^ in children and 95.25 mg·h L^−1^ in adults [103].

The recommended dosing regimen in pediatric patients older than one month is a loading dose of 3 mg/kg/day followed by 1.5 mg/kg daily [98,99].

### 6.2. Efficacy

The available data on the efficacy of anidulafungin in infants with IC are limited. However, the drug has been demonstrated to be effective against various *Candida* species in adult populations [103]. The unique metabolic pathway of the drug necessitates no dose adjustment in cases of renal or hepatic impairment, which may prove beneficial in the NICU population, particularly in high-risk neonates with a high prevalence of comorbidities.

A major concern in the neonatal IC treatment with anidulafungin is the high rate of CNS involvement, a site where penetration of echinocandins is uncertain [103]. A bridging study using a rabbit model of HCME demonstrated that the penetration of the drug into brain tissue is dose-dependent and suggested that higher doses of the drug than those used to produce a similar exposure to that of adults of anidulafungin are required for neonates, given the high rate of CNS involvement [106]. Dose-dependent CNS penetration has also been demonstrated with other echinocandins [58].

Roilides et al. reported an 81.2% (13/16) successful *Candida* spp. eradication in a population of infants <2 years of age with culture-confirmed IC treated with anidulafungin. Of the patients with persistently positive cultures, two were infected with candidemia caused by *C. albicans* and one with *C. parapsilosis* [107]. The same group of researchers reported an efficacy of 77.8% at the end of treatment in patients aged 2 to 5 years and 66.7% in older children and adolescents [108]. In both studies, the median time to negative cultures was three days after anidulafungin initiation [107,108].

**Table 4:** Clinical studies evaluating the pharmacokinetics of anidulafungin in pediatric populations.

Author	Population	Dose	Clearance (L/kg/h)	AUC_0–24_ (mg·h L^−1^)	T1/2 ()
Benjamin, 2006 [104]	12 patients (2–11 years old)	1.5 mg/kg loading dose, 0.75 mg/kg maintenance	0.0217 (0.0113–0.0446)	41.1 (16.5–57.8)	20.3 (13.9–35.1)
		3 mg/kg loading dose, 1.5 mg/kg maintenance	0.0133 (0.0095–0.018)	96.1 (43.2–155.7)	26.0 (12.0–38.9)
	13 patients (12–17 years old)	1.5 mg/kg loading dose, 0.75 mg/kg maintenance	0.0163 (0.0094–0.0231)	56.2 (31.8–79.8)	18.9 (13.6–24.1)
		3 mg/kg loading dose, 1.5 mg/kg maintenance	0.0156 (0.0096–0.0311)	102.9 (50.3–134.1)	21.1 (15.0–27.8)
Cohen-Wolkoviez, 2012 [105]	8 neonates (<30 days)	3 mg/kg loading dose, 1.5 mg/kg day maintenance	0.020 (0.013–0.049)	74.9 (30.4–108.9)	78 (40–219)
	7 infants (>30 days)		0.015 (0.005–0.027)	97.7 (54.8–278.0)	33 (30–173)
Roilides, 2020 [107]	19 infants (1 month–2 years old)	3 mg/kg loading dose, 1.5 mg/kg day maintenance	No data	70.2 (42.9–87.7)	24.0 (23.7–24.4)

### 6.3. Adverse Effects

Anidulafungin has been shown to be well tolerated in the pediatric population with mild and transient adverse events that rarely necessitate treatment discontinuation. The most commonly reported side effects include gastrointestinal disturbances, fever, rash, and elevation of liver enzymes [104,105,106,107,108]. Infusion-related reactions such as flushing, rash, dyspnea, and hypotension have been reported, and an infusion rate of less than 1.1 mg/min is recommended [98,99,104].

A notable concern regarding the administration of anidulafungin in neonates is the uncertainty regarding the potential accumulation of polysorbate 80 (PS80) [99]. Polysorbates are surfactants widely used in therapeutic formulations and are considered safe within established exposure limits. However, the immaturity of the detoxification systems in children, particularly in preterm neonates, renders them more vulnerable, and the safe limits of exposure are yet to be defined [109,110]. PS80 levels following anidulafungin administration to infants have been evaluated in one study. No cases of PS80 accumulation or hepatotoxicity were reported [107].

## 7. Special Clinical Conditions

### 7.1. Candida Auris Treatment

*Candida auris*, first isolated in Japan in 2009, is an emerging pathogen of growing global concern due to its rapid spread in the hospital environment and its multidrug-resistant profile. A particular concern in NICUs is that *C. auris* strains are generally resistant to conventional antifungal agents administered to neonates, fluconazole, and amphotericin [31,40,111,112]. *C. auris* isolates were resistant to fluconazole in 97.4% and to amphotericin in 67.1%, according to a recent systematic review [111]. Echinocandins have been demonstrated to be effective against this multidrug-resistant strain, although resistance has been documented in some cases [31,111]. According to the recommendations of the Centers for Disease and Prevention (CDC), echinocandins are the drug of choice for *C. auris* infection, excluding neonates. However, in certain circumstances, echinocandin therapy may be considered for neonates after CNS involvement has been excluded [113].

Chandramati et al. recently reported an outbreak of 17 cases of *C. auris* infection in a NICU in India. All isolates were susceptible to micafungin, resistant to fluconazole, and intermediately susceptible to amphotericin. The overall mortality rate was 41%. Among these patients, 86% had not received micafungin. Once the susceptibility patterns were known, treatment with micafungin was initiated, resulting in a 91% survival rate. Moreover, the use of micafungin instead of fluconazole as prophylaxis in high-risk neonates resulted in a significant reduction in the *C. auris* infection rate from 6.25 to 0.8% [68]. However, the management of systemic *C. auris* infection is challenging, especially in the most vulnerable preterm neonates. In a small cohort of preterm neonates with less than 800 g birth weight and with *C. auris* susceptible to micafungin, 80% (4/5) died despite micafungin administration [114].

### 7.2. Activity Against Biofilms

The formation of biofilms, which are three-dimensional structures of fungal cells organized within an extracellular polymeric matrix that adheres to either host tissue or implanted medical devices, is an important aspect of the pathogenesis of *Candida* spp. infections [115,116,117]. Biofilm formation is associated with significant morbidity and mortality rates because the fungi within these structures remain protected from antifungal agents and the host immune response, and biofilms also act as sites for continued fungal dissemination [118,119]. Furthermore, the development of antifungal resistance is a well-documented characteristic of *Candida* biofilms. Possible contributors to this phenomenon include increased metabolic activity in the early stages of biofilm formation, the barrier provided by the extracellular polymeric matrix of biofilms, and changes in gene expression [120]. Echinocandins have been demonstrated to be potent against *Candida* biofilms, exerting both anti-adherent and antibiofilm activity even at low doses [121]. In addition, echinocandins have been shown to act synergistically with neutrophils against *Candida* biofilms by enhancing their activity and promoting the formation of neutrophil extracellular traps [118,122].

Biofilm formation on implanted medical devices such as central venous catheters (CVCs), routinely used in premature infants for nutrition and medical support, poses a significant therapeutic challenge in NICU patients. In neonatal systemic candidiasis, prompt CVC removal has been associated with improved survival and is strongly recommended [10,123]. However, this is not always feasible, especially in critically ill VLBW neonates [117]. In instances where vascular catheters cannot be removed, systemic antifungal treatment is often insufficient to achieve *Candida* eradication, and lock therapy may be considered [121].

Lock therapy is a procedure that involves the diffusion and retention of a high dose of an antifungal agent within a vascular catheter lumen [120,121,124]. A recent review and meta-analysis in pediatric patients with catheter-related or catheter-associated bloodstream infections (CRSBI or CLABSI) concluded that the combination of systemic and lock antimicrobial therapy was more effective than systemic antimicrobials alone in achieving catheter salvage and reducing the recurrence of infection in CRSBIs. However, no clear advantage was observed with the addition of lock therapy to systemic antimicrobial therapy in CLASBIs [125]. Among the antifungals, lock therapy with echinocandins and lipid formulations of amphotericin have been shown to be effective [121,126]. The efficacy of lock therapy with all three echinocandins against a broad range of *Candida* spp. has been demonstrated in several studies [121,124]. In their investigation, Petraitiene et al. evaluated the efficacy of systemic and lock micafungin in experimental animals. They concluded that the combination of the two strategies was the most effective approach for systemic candidemia when catheter removal was not feasible, as systemic therapy alone could not achieve catheter eradication and lock therapy was insufficient in treating deep tissue infection [32]. Micafungin has been shown to have a concentration-dependent activity against *Candida* spp., with higher doses diffused in the catheter being more effective [32,126].

Nevertheless, a number of in vitro and animal experimental studies have highlighted the potential for a paradoxical effect associated with echinocandins, particularly with regard to caspofungin. The paradoxical effect is defined as a reduction in the susceptibility of certain *Candida* spp. to the drug at higher doses compared to lower doses [127]. This phenomenon has been documented in several studies where high doses of caspofungin were used as lock therapy to treat *Candida* biofilms. However, the clinical significance of this remains a topic of debate [127,128,129,130,131].

## 8. Optimizing Empiric Antifungal Use in the NICU

The prompt initiation of antifungal treatment in neonates has been associated with improved neurodevelopmental outcomes and survival rates. The lack of specific clinical manifestations of IC and the diagnostic limitations, including the low sensitivity and long turnaround time of blood cultures and the lack of reliable biomarkers, often necessitate the empirical administration of antifungal agents in high-risk neonates [132]. This results in a significant discrepancy between the number of infants treated with empirical antifungals and those with a proven fungal infection [133]. A recent multicenter study demonstrated that less than a quarter of neonates treated with empirical antifungals eventually had a confirmed diagnosis of IC [134]. Furthermore, a population-based German multicenter study revealed that 5.4% of VLBW infants were exposed to antifungal agents, although only 0.3% had a confirmed fungal infection [133].

Although empirical treatment administration in high-risk neonates cannot be avoided due to the inability in most settings to exclude *Candida* infection promptly and accurately, clinicians should aim to optimize empirical antifungal therapy by selecting the most appropriate agent and ensuring its timely discontinuation.

The rising prevalence of non-*albicans Candida* spp. and the widespread use of fluconazole prophylaxis in NICUs have contributed to an increase in the incidence of isolates resistant to first-line antifungal agents [132]. The selection of an antifungal agent for empirical treatment should be made in accordance with the strategies for antifungal prophylaxis employed in the NICU and the local resistance pattern. However, the identification of the causative strain and susceptibility pattern is critical for effective, targeted treatment. The implementation of rapid and accurate diagnostic techniques provides the opportunity to minimize the empirical administration of antifungals and to initiate targeted therapy directed at the isolated fungi.

Another issue regarding the empirical antifungal treatment in neonates is that it is often continued despite the presence of sterile cultures [133]. A number of studies have demonstrated that the reduction in antibiotic exposure, achieved by discontinuing antimicrobial therapy following negative cultures, can lead to improved outcomes for preterm and very low birth weight neonates [135,136,137]. The utilization of short antimicrobial courses in the absence of evidence of infection has been demonstrated to be an efficacious strategy in the NICU, with no discernible increase in treatment failure in instances of true infection [138]. The same approach of administering short courses of antifungals in cases of unproven fungal infection should optimize the use of antifungals in the NICU, minimizing adverse effects and the emergence of resistance [134,139,140].

The aforementioned strategies represent the fundamental aspects of antifungal stewardship programs (AFSs), which are designed to facilitate the rational and appropriate utilization of antifungals [134,139,141].

## 9. Limitations

As anticipated, given the narrative nature of this review, there are some limitations, including the non-standardized literature search, which may lead to selection bias. Furthermore, only articles written in English were used in the preparation of this narrative review. However, an extensive literature search was conducted, minimizing the potential impact of the above limitations.

## 10. Conclusions

The range of therapeutic options for the treatment of invasive candidiasis has been expanded in recent decades due to the availability of new antifungal agents. However, extrapolating data from adult studies to the neonatal population is an unsuitable approach, and studies in neonates are scarce, preventing the recommendation of the use of several new antifungal agents in this population.

Echinocandins share characteristics that render them a reasonable choice as an antifungal agent in the NICU, including a broad spectrum of activity, activity against biofilms, and a favorable safety profile. Among the echinocandins, micafungin is the most extensively studied and is currently the only one approved for use in neonatal IC. However, there is growing evidence from recent literature that caspofungin and anidulafungin are also safe and effective in the treatment of systemic candidiasis in neonates.

Nevertheless, despite the potential therapeutic benefits of echinocandins in NICUs, their widespread use is not currently recommended due to the limited evidence based on this population. Future, larger, prospective, controlled trials in neonatal populations are needed to provide evidence on the pharmacokinetics, efficacy, and tolerability of these agents in this population before they can be incorporated into the guidelines for neonatal IC treatment.

## Figures and Tables

**Table 1:** The literature search strategy.

Database	PubMed, Google Scholar
Keywords	neonatal invasive candidiasis, preterm neonate, candidiasis treatment, antifungal agents, echinocandins, micafungin, caspofungin, anidulafungin
Timeframe	Up to 10 October 2024
Inclusion and exclusion criteria	English, full-text articles

## Data Availability

No new data were created or analyzed in this study.

## References

[1] Weimer K.E.D., Smith P.B., Puia-Dumitrescu M., Aleem S. (2022). Invasive fungal infections in neonates: A review. Pediatr. Res..

[2] Flannery D.D., Edwards E.M., Coggins S.A., Horbar J.D., Puopolo K.M. (2022). Late-Onset Sepsis Among Very Preterm Infants. Pediatrics.

[3] Zhou Q., Kelly E., Luu T.M., Ye X.Y., Ting J., Shah P.S., Lee S.K. (2023). Fungal infection and neurodevelopmental outcomes at 18–30 months in preterm infants. Front. Pediatr..

[4] Bersani I., Piersigilli F., Goffredo B.M., Santisi A., Cairoli S., Ronchetti M.P., Auriti C. (2019). Antifungal Drugs for Invasive Candida Infections (ICI) in Neonates: Future Perspectives. Front. Pediatr..

[5] Benjamin D.K., Stoll B.J., Fanaroff A.A., McDonald S.A., Oh W., Higgins R.D., Duara S., Poole K., Laptook A., Goldberg R. (2006). National Institute of Child Health and Human Development Neonatal Research Network. Neonatal candidiasis among extremely low birth weight infants: Risk factors, mortality rates, and neurodevelopmental outcomes at 18 to 22 months. Pediatrics.

[6] Han T., Qiu M., Niu X., Wang S., Wang F., Cao J., Tang S., Cheng L., Mei Y., Liang H. (2024). End-organ damage from neonatal invasive fungal infection: A 14-year retrospective study from a tertiary center in China. BMC Infect. Dis..

[7] Pammi M., Edwards M., Puopolo K. (2024). Candida Infections in Neonates: Epidemiology, Clinical Manifestations, and Diagnosis.

[8] Adams-Chapman I., Bann C.M., Das A., Goldberg R.N., Stoll B.J., Walsh M.C., Sánchez P.J., Higgins R.D., Shankaran S., Watterberg K.L. (2013). Neurodevelopmental outcome of extremely low birth weight infants with Candida infection. J. Pediatr..

[9] Taormina G., Gopinath R., Moore J., Yasinskaya Y., Colangelo P., Reynolds K., Nambiar S. (2021). A Regulatory Review Approach for Evaluation of Micafungin for Treatment of Neonatal Candidiasis. Clin. Infect. Dis..

[10] Pappas P.G., Kauffman C.A., Andes D.R., Clancy C.J., Marr K.A., Ostrosky-Zeichner L., Reboli A.C., Schuster M.G., Vazquez J.A., Walsh T.J. (2016). Clinical Practice Guideline for the Management of Candidiasis: 2016 Update by the Infectious Diseases Society of America. Clin. Infect. Dis..

[11] Ting J.Y., Roberts A., Synnes A., Canning R., Bodani J., Monterossa L., Shah P.S. (2018). Canadian Neonatal Network Investigators. Invasive Fungal Infections in Neonates in Canada: Epidemiology and Outcomes. Pediatr. Infect. Dis. J..

[12] Warris A., Pana Z.D., Oletto A., Lundin R., Castagnola E., Lehrnbecher T., Groll A.H., Roilides E. (2020). Eurocandy Study Group. Etiology and Outcome of Candidemia in Neonates and Children in Europe: An 11-year Multinational Retrospective Study. Pediatr. Infect. Dis. J..

[13] Benedict K., Roy M., Kabbani S., Anderson E.J., Farley M.M., Harb S., Harrison L.H., Bonner L., Wadu V.L., Marceaux K. (2018). Neonatal and Pediatric Candidemia: Results from Population-Based Active Laboratory Surveillance in Four US Locations, 2009–2015. J. Pediatr. Infect. Dis. Soc..

[14] Chakrabarti A., Sood P., Rudramurthy S.M., Chen S., Jillwin J., Iyer R., Sharma A., Harish B.N., Roy I., Kindo A.J. (2020). Characteristics, outcome and risk factors for mortality of paediatric patients with ICU-acquired candidemia in India: A multicentre prospective study. Mycoses.

[15] Cook A., Ferreras-Antolin L., Adhisivam B., Ballot D., Berkley J.A., Bernaschi P., Carvalheiro C.G., Chaikittisuk N., Chen Y., Chibabhai V. (2023). Neonatal invasive candidiasis in low- and middle-income countries: Data from the NeoOBS study. Med. Mycol..

[16] Noni M., Stathi A., Vaki I., Velegraki A., Zachariadou L., Michos A. (2019). Changing Epidemiology of Invasive Candidiasis in Children during a 10-Year Period. J. Fungi.

[17] He B., Yang Q. (2023). Updates in Laboratory Identification of Invasive Fungal Infection in Neonates. Microorganisms.

[18] Daniel K., Greenberg R.G., Boutzoukas A., Katakam L. (2023). Updated Perspectives on the Diagnosis and Management of Neonatal Invasive Candidiasis. Res. Rep. Neonatol..

[19] Wang K., Luo Y., Zhang W., Xie S., Yan P., Liu Y., Li Y., Ma X., Xiao K., Fu H. (2020). Diagnostic value of Candida mannan antigen and anti-mannan IgG and IgM antibodies for Candida infection. Mycoses.

[20] Ferreras-Antolin L., Borman A., Diederichs A., Warris A., Lehrnbecher T. (2022). Serum Beta-D-Glucan in the Diagnosis of Invasive Fungal Disease in Neonates, Children and Adolescents: A Critical Analysis of Current Data. J. Fungi.

[21] Camp I., Spettel K., Willinger B. (2020). Molecular Methods for the Diagnosis of Invasive Candidiasis. J. Fungi.

[22] Turkova A., Roilides E., Sharland M. (2011). Amphotericin B in neonates: Deoxycholate or lipid formulation as first-line therapy—Is there a ‘right’ choice?. Curr. Opin. Infect. Dis..

[23] Akinosoglou K., Rigopoulos E.A., Papageorgiou D., Schinas G., Polyzou E., Dimopoulou E., Gogos C., Dimopoulos G. (2024). Amphotericin B in the Era of New Antifungals: Where Will It Stand?. J. Fungi.

[24] Scott B.L., Hornik C.D., Zimmerman K. (2020). Pharmacokinetic, efficacy, and safety considerations for the use of antifungal drugs in the neonatal population. Expert. Opin. Drug Metab. Toxicol..

[25] Hornik C.D., Bondi D.S., Greene N.M., Cober M.P., John B. (2021). Review of Fluconazole Treatment and Prophylaxis for Invasive Candidiasis in Neonates. J. Pediatr. Pharmacol. Ther..

[26] Carmo A., Rocha M., Pereirinha P., Tomé R., Costa E. (2023). Antifungals: From Pharmacokinetics to Clinical Practice. Antibiotics.

[27] Sigera L.S.M., Denning D.W. (2023). Flucytosine and its clinical usage. Ther. Adv. Infect. Dis..

[28] Hope W.W., Castagnola E., Groll A.H., Roilides E., Akova M., Arendrup M.C., Arikan-Akdagli S., Bassetti M., Bille J., Cornely O.A. (2012). ESCMID* guideline for the diagnosis and management of Candida diseases 2012: Prevention and management of invasive infections in neonates and children caused by *Candida* spp.. Clin. Microbiol. Infect..

[29] Faustino C., Pinheiro L. (2020). Lipid Systems for the Delivery of Amphotericin B in Antifungal Therapy. Pharmaceutics.

[30] Tezer H., Canpolat F.E., Dilmen U. (2012). Invasive fungal infections during the neonatal period: Diagnosis, treatment and prophylaxis. Expert. Opin. Pharmacother..

[31] Lone S.A., Ahmad A. (2019). Candida auris-the growing menace to global health. Mycoses.

[32] Petraitiene R., Petraitis V., Zaw M.H., Hussain K., Ricart Arbona R.J., Roilides E., Walsh T.J. (2024). Combination of Systemic and Lock-Therapies with Micafungin Eradicate Catheter-Based Biofilms and Infections Caused by Candida albicans and Candida parapsilosis in Neutropenic Rabbit Models. J. Fungi.

[33] Aguilar-Zapata D., Petraitiene R., Petraitis V. (2015). Echinocandins: The Expanding Antifungal Armamentarium. Clin. Infect. Dis..

[34] Denning D.W. (2003). Echinocandin antifungal drugs. Lancet.

[35] Szymański M., Chmielewska S., Czyżewska U., Malinowska M., Tylicki A. (2022). Echinocandins-structure, mechanism of action and use in antifungal therapy. J. Enzym. Inhib. Med. Chem..

[36] Mroczyńska M., Brillowska-Dąbrowska A. (2020). Review on Current Status of Echinocandins Use. Antibiotics.

[37] Chen S.C., Slavin M.A., Sorrell T.C. (2011). Echinocandin antifungal drugs in fungal infections: A comparison. Drugs.

[38] Jauregizar N., Quindós G., Gil-Alonso S., Suárez E., Sevillano E., Eraso E. (2022). Postantifungal Effect of Antifungal Drugs against *Candida*: What Do We Know and How Can We Apply This Knowledge in the Clinical Setting?. J. Fungi.

[39] Prayag P.S., Patwardhan S.A., Joshi R.S., Dhupad S., Rane T., Prayag A.P. (2024). Comparative efficacies of the three echinocandins for Candida auris candidemia: Real world evidence from a tertiary centre in India. Med. Mycol..

[40] Bandara N., Samaranayake L. (2022). Emerging and future strategies in the management of recalcitrant Candida auris. Med. Mycol..

[41] Lestner J.M., Smith P.B., Cohen-Wolkowiez M., Benjamin D.K., Hope W.W. (2013). Antifungal agents and therapy for infants and children with invasive fungal infections: A pharmacological perspective. Br. J. Clin. Pharmacol..

[42] Wiederhold N.P. (2016). Echinocandin Resistance in Candida Species: A Review of Recent Developments. Curr. Infect. Dis. Rep..

[43] Sherry L., Ramage G., Kean R., Borman A., Johnson E.M., Richardson M.D., Rautemaa-Richardson R. (2017). Biofilm-Forming Capability of Highly Virulent, Multidrug-Resistant Candida auris. Emerg. Infect. Dis..

[44] Tsekoura M., Ioannidou M., Pana Z.D., Haidich A.B., Antachopoulos C., Iosifidis E., Kolios G., Roilides E. (2019). Efficacy and Safety of Echinocandins for the Treatment of Invasive Candidiasis in Children: A Meta-analysis. Pediatr. Infect. Dis. J..

[45] Kauffman C.A., Carver P.L. (2008). Update on echinocandin antifungals. Semin. Respir. Crit. Care Med..

[46] Chen Y.H., Cheng I.L., Lai C.C., Tang H.J. (2019). Echinocandins vs. amphotericin B against invasive candidiasis in children and neonates: A meta-analysis of randomized controlled trials. Int. J. Antimicrob. Agents..

[47] Food and Drug Administration (FDA). https://www.accessdata.fda.gov/drugsatfda_docs/label/2019/021506s023lbl.pdf.

[48] European Medicines Agency (EMA). https://www.ema.europa.eu/en/documents/product-information/mycamine-epar-product-information_en.pdf.

[49] De Rose D.U., Bersani I., Ronchetti M.P., Piersigilli F., Cairoli S., Dotta A., Desai A., Kovanda L.L., Goffredo B.M., Auriti C. (2023). Plasma and Cerebrospinal Fluid Concentrations of Micafungin Administered at High Doses in Critically Ill Infants with Systemic Candidiasis: A Pooled Analysis of Two Studies. Pharmaceuticals.

[50] Ruggiero A., Ariano A., Triarico S., Capozza M.A., Ferrara P., Attinà G. (2019). Neonatal pharmacology and clinical implications. Drugs Context..

[51] Yanni S.B., Smith P.B., Benjamin D.K.J., Augustijns P.F., Thakker D.R., Annaert P.P. (2011). Higher clearance of micafungin in neonates compared with adults: Role of age-dependent micafungin serum binding. Biopharm. Drug Dispos..

[52] Hope W.W., Kaibara A., Roy M., Arrieta A., Azie N., Kovanda L.L., Benjamin D.K. (2015). Population pharmacokinetics of micafungin and its metabolites M1 and M5 in children and adolescents. Antimicrob. Agents Chemother..

[53] Smith P.B., Walsh T.J., Hope W., Arrieta A., Takada A., Kovanda L.L., Kearns G.L., Kaufman D., Sawamoto T., Buell D.N. (2009). Pharmacokinetics of an elevated dosage of micafungin in premature neonates. Pediatr. Infect. Dis. J..

[54] Heresi G.P., Gerstmann D.R., Reed M.D., van den Anker J.N., Blumer J.L., Kovanda L., Keirns J.J., Buell D.N., Kearns G.L. (2006). The pharmacokinetics and safety of micafungin, a novel echinocandin, in premature infants. Pediatr. Infect. Dis. J..

[55] Benjamin D.K., Smith P.B., Arrieta A., Castro L., Sánchez P.J., Kaufman D., Arnold L.J., Kovanda L.L., Sawamoto T., Buell D.N. (2010). Safety and pharmacokinetics of repeat-dose micafungin in young infants. Clin. Pharmacol. Ther..

[56] Benjamin D.K., Kaufman D.A., Hope W.W., Smith P.B., Arrieta A., Manzoni P., Kovanda L.L., Lademacher C., Isaacson B., Jednachowski D. (2018). A Phase 3 Study of Micafungin Versus Amphotericin B Deoxycholate in Infants With Invasive Candidiasis. Pediatr. Infect. Dis. J..

[57] Leroux S., Jacqz-Aigrain E., Elie V., Legrand F., Barin-Le Guellec C., Aurich B., Biran V., Dusang B., Goudjil S., Coopman S. (2018). Pharmacokinetics and safety of fluconazole and micafungin in neonates with systemic candidiasis: A randomized, open-label clinical trial. Br. J. Clin. Pharmacol..

[58] Hope W.W., Mickiene D., Petraitis V., Petraitiene R., Kelaher A.M., Hughes J.E., Cotton M.P., Bacher J., Keirns J.J., Buell D. (2008). The pharmacokinetics and pharmacodynamics of micafungin in experimental hematogenous Candida meningoencephalitis: Implications for echinocandin therapy in neonates. J. Infect. Dis..

[59] Parramon-Teixido C.J., Garcia Esquerda C., Frick M.A., Tripodi C., Gomez-Ganda L., Ruiz-Campillo C.W., Cabañas-Poy M.J. (2024). Case Report: Micafungin for treating *Candida glabrata*, urinary infection: A clinical case in a premature neonate. Front. Pediatr..

[60] Grau S., Luque S., Echeverría-Esnal D., Sorlí L., Campillo N., Montero M., Álvarez Lerma F., Plasencia V., Horcajada J.P. (2016). Urinary micafungin levels are sufficient to treat urinary tract infections caused by *Candida* spp.. Int. J. Antimicrob. Agents..

[61] Kane L.E., Muzevich K.M. (2016). Micafungin in the treatment of candiduria: A case series. Med. Mycol. Case Rep..

[62] Hope W.W., Smith P.B., Arrieta A., Buell D.N., Roy M., Kaibara A., Walsh T.J., Cohen-Wolkowiez M., Benjamin D.K. (2010). Population pharmacokinetics of micafungin in neonates and young infants. Antimicrob. Agents Chemother..

[63] Javorova Rihova Z., Slobodova L., Hrabovska A. (2021). Micafungin Is an Efficient Treatment of Multi Drug-Resistant *Candida glabrata* Urosepsis: A Case Report. J. Fungi.

[64] Auriti C., Goffredo B.M., Ronchetti M.P., Piersigilli F., Cairoli S., Bersani I., Dotta A., Bagolan P., Pai M.P. (2021). High-Dose Micafungin in Neonates and Young Infants with Invasive Candidiasis: Results of a Phase 2 Study. Antimicrob. Agents Chemother..

[65] Chiotos K., Vendetti N., Zaoutis T.E., Baddley J., Ostrosky-Zeichner L., Pappas P., Fisher B.T. (2016). Comparative effectiveness of echinocandins versus fluconazole therapy for the treatment of adult candidaemia due to Candida parapsilosis: A retrospective observational cohort study of the Mycoses Study Group (MSG-12). J. Antimicrob. Chemother..

[66] De Rose D.U., Piersigilli F., Goffredo B.M., Danhaive O., Dotta A., Auriti C. (2021). Treatment with Micafungin in a Preterm Neonate with an Invasive *Candida parapsilosis* Infection after a Severe Terlipressin-Induced Skin Necrosis. Pathogens.

[67] Khodavaisy S., Badali H., Meis J.F., Modiri M., Mahmoudi S., Abtahi H., Salehi M., Manshadi S.A.D., Aala F., Afshari S.A.K. (2020). Comparative in vitro activities of seven antifungal drugs against clinical isolates of Candida parapsilosiscomplex. J. Mycol. Med..

[68] Chandramati J., Sadanandan L., Kumar A., Ponthenkandath S. (2020). Neonatal Candida auris infection: Management and prevention strategies—A single centre experience. J. Paediatr. Child. Health.

[69] Auriti C., Falcone M., Ronchetti M.P., Goffredo B.M., Cairoli S., Crisafulli R., Piersigilli F., Corsetti T., Dotta A., Pai M.P. (2016). High-Dose Micafungin for Preterm Neonates and Infants with Invasive and Central Nervous System Candidiasis. Antimicrob. Agents Chemother..

[70] Manzoni P., Wu C., Tweddle L., Roilides E. (2014). Micafungin in premature and non-premature infants: A systematic review of 9 clinical trials. Pediatr. Infect. Dis. J..

[71] King K.Y., Edwards M.S., Word B.M. (2009). Hepatitis associated with micafungin use in a preterm infant. J. Perinatol..

[72] Wasmann R.E., Muilwijk E.W., Burger D.M., Verweij P.E., Knibbe C.A., Brüggemann R.J. (2018). Clinical Pharmacokinetics and Pharmacodynamics of Micafungin. Clin. Pharmacokinet..

[73] Undre N.A., Stevenson P., Wilbraham D. (2014). Pharmacokinetic profile of micafungin when co-administered with amphotericin B in healthy male subjects. Int. J. Clin. Pharmacol. Ther..

[74] Kofla G., Ruhnke M. (2011). Pharmacology and metabolism of anidulafungin, caspofungin and micafungin in the treatment of invasive candidosis: Review of the literature. Eur. J. Med. Res..

[75] Hashemian S.M., Farhadi T., Velayati A.A. (2020). Caspofungin: A review of its characteristics, activity, and use in intensive care units. Expert. Rev. Anti Infect. Ther..

[76] Walsh T.J., Adamson P.C., Seibel N.L., Flynn P.M., Neely M.N., Schwartz C., Shad A., Kaplan S.L., Roden M.M., Stone J.A. (2005). Pharmacokinetics, safety, and tolerability of caspofungin in children and adolescents. Antimicrob. Agents Chemother..

[77] Neely M., Jafri H.S., Seibel N., Knapp K., Adamson P.C., Bradshaw S.K., Strohmaier K.M., Sun P., Bi S., Dockendorf M.F. (2009). Pharmacokinetics and safety of caspofungin in older infants and toddlers. Antimicrob. Agents Chemother..

[78] Sáez-Llorens X., Macias M., Maiya P., Pineros J., Jafri H.S., Chatterjee A., Ruiz G., Raghavan J., Bradshaw S.K., Kartsonis N.A. (2009). Pharmacokinetics and safety of caspofungin in neonates and infants less than 3 months of age. Antimicrob. Agents Chemother..

[79] Food and Drug Administration (FDA). https://www.accessdata.fda.gov/drugsatfda_docs/label/2016/206110lbl.pdf.

[80] Wiederhold N.P., Herrera L.A. (2012). Caspofungin for the treatment of immunocompromised and severely ill children and neonates with invasive fungal infections. Clin. Med. Insights Pediatr..

[81] Marion D., Caroline S., Renaud V., Romain G. (2022). Advocacy for close monitoring of caspofungin therapy in premature infants: A case report. Pediatr. Neonatol..

[82] Yang X.M., Leroux S., Storme T., Zhang D.L., de Beaumais T.A., Shi H.Y., Yang Y.L., Wang X.L., Zhao W., Jacqz-Aigrain E. (2019). Body Surface Area-Based Dosing Regimen of Caspofungin in Children: A Population Pharmacokinetics Confirmatory Study. Antimicrob. Agents Chemother..

[83] Li C.C., Sun P., Dong Y., Bi S., Desai R., Dockendorf M.F., Kartsonis N.A., Ngai A.L., Bradshaw S., Stone J.A. (2011). Population pharmacokinetics and pharmacodynamics of caspofungin in pediatric patients. Antimicrob. Agents Chemother..

[84] Mori M., Imaizumi M., Ishiwada N., Kaneko T., Goto H., Kato K., Hara J., Kosaka Y., Koike K., Kawamoto H. (2015). Pharmacokinetics, efficacy, and safety of caspofungin in Japanese pediatric patients with invasive candidiasis and invasive aspergillosis. J. Infect. Chemother..

[85] Zaoutis T.E., Jafri H.S., Huang L.M., Locatelli F., Barzilai A., Ebell W., Steinbach W.J., Bradley J., Lieberman J.M., Hsiao C.C. (2009). A prospective, multicenter study of caspofungin for the treatment of documented Candida or Aspergillus infections in pediatric patients. Pediatrics.

[86] Niu C.H., Xu H., Gao L.L., Nie Y.M., Xing L.P., Yu L.P., Wu S.L., Wang Y. (2020). Population Pharmacokinetics of Caspofungin and Dosing Optimization in Children with Allogeneic Hematopoietic Stem Cell Transplantation. Front. Pharmacol..

[87] Kim J., Nakwa F.L., Araujo Motta F., Liu H., Dorr M.B., Anderson L.J., Kartsonis N. (2020). A randomized, double-blind trial investigating the efficacy of caspofungin versus amphotericin B deoxycholate in the treatment of invasive candidiasis in neonates and infants younger than 3 months of age. J. Antimicrob. Chemother..

[88] Jans J., Brüggemann R.J., Christmann V., Verweij P.E., Warris A. (2013). Favorable outcome of neonatal cerebrospinal fluid shunt-associated Candida meningitis with caspofungin. Antimicrob. Agents Chemother..

[89] Smith P.B., Steinbach W.J., Cotten C.M., Schell W.A., Perfect J.R., Walsh T.J., Benjamin D.K. (2007). Caspofungin for the treatment of azole resistant candidemia in a premature infant. J. Perinatol..

[90] Al-Sweih N., Ahmad S., Khan S., Khan Z., Joseph L., Vayalil S., Chandy R. (2017). Persistent Candida conglobata bloodstream infection in a preterm neonate successfully treated by combination therapy with amphotericin B and caspofungin. J. Mycol. Med..

[91] Natale F., Castronovo A., Regoli D., De Curtis M., Manzoni P. (2009). Successful treatment with caspofungin of refractory Candida krusei candidemia in a very low birth weight preterm infant. Pediatr. Infect. Dis. J..

[92] Manzar S., Kamat M., Pyati S. (2006). Caspofungin for refractory candidemia in neonates. Pediatr. Infect. Dis. J..

[93] Natarajan G., Lulic-Botica M., Rongkavilit C., Pappas A., Bedard M. (2005). Experience with caspofungin in the treatment of persistent fungemia in neonates. J. Perinatol..

[94] Jeon G.W., Sin J.B. (2014). Successful caspofungin treatment of persistent candidemia in extreme prematurity at 23 and 24 weeks’ gestation. J. Formos. Med. Assoc..

[95] Odio C.M., Araya R., Pinto L.E., Castro C.E., Vasquez S., Alfaro B., Sàenz A., Herrera M.L., Walsh T.J. (2004). Caspofungin therapy of neonates with invasive candidiasis. Pediatr. Infect. Dis. J..

[96] Mohamed W.A., Ismail M. (2012). A randomized, double-blind, prospective study of caspofungin vs. amphotericin B for the treatment of invasive candidiasis in newborn infants. J. Trop. Pediatr..

[97] Autmizguine J., Guptill J.T., Cohen-Wolkowiez M., Benjamin D.K., Capparelli E.V. (2014). Pharmacokinetics and pharmacodynamics of antifungals in children: Clinical implications. Drugs.

[98] European Medicines Agency (EMA). https://www.ema.europa.eu/en/documents/product-information/ecalta-epar-product-information_en.pdf.

[99] Food and Drug Administration (FDA). https://www.accessdata.fda.gov/drugsatfda_docs/label/2020/021632027_S029lbl.pdf.

[100] Marx J., Welte R., Gasperetti T., Moser P., Joannidis M., Bellmann R. (2021). Human Tissue Distribution of Anidulafungin and Micafungin. Antimicrob. Agents Chemother..

[101] Marx J., Welte R., Gasperetti T., Moser P., Beer R., Ortler M., Jeske M., Stern R., Pomaroli A., Joannidis M. (2020). Anidulafungin and Micafungin Concentrations in Cerebrospinal Fluid and in Cerebral Cortex. Antimicrob. Agents Chemother..

[102] Ripp S.L., Aram J.A., Bowman C.J., Chmielewski G., Conte U., Cross D.M., Gao H., Lewis E.M., Lin J., Liu P. (2012). Tissue distribution of anidulafungin in neonatal rats. Birth Defects Res. B Dev. Reprod. Toxicol..

[103] Xie R., McFadyen L., Raber S., Swanson R., Tawadrous M., Leister-Tebbe H., Cohen-Wolkowiez M., Benjamin D.K., Liu P. (2020). Population Analysis of Anidulafungin in Infants to Older Adults with Confirmed or Suspected Invasive Candidiasis. Clin. Pharmacol. Ther..

[104] Benjamin D.K., Driscoll T., Seibel N.L., Gonzalez C.E., Roden M.M., Kilaru R., Clark K., Dowell J.A., Schranz J., Walsh T.J. (2006). Safety and pharmacokinetics of intravenous anidulafungin in children with neutropenia at high risk for invasive fungal infections. Antimicrob. Agents Chemother..

[105] Cohen-Wolkowiez M., Benjamin D.K., Piper L., Cheifetz I.M., Moran C., Liu P., Aram J., Kashuba A.D., Capparelli E., Walsh T.J. (2011). Safety and pharmacokinetics of multiple-dose anidulafungin in infants and neonates. Clin. Pharmacol. Ther..

[106] Warn P.A., Livermore J., Howard S., Felton T.W., Sharp A., Gregson L., Goodwin J., Petraitiene R., Petraitis V., Cohen-Wolkowiez M. (2012). Anidulafungin for neonatal hematogenous Candida meningoencephalitis: Identification of candidate regimens for humans using a translational pharmacological approach. Antimicrob. Agents Chemother..

[107] Roilides E., Carlesse F., Tawadrous M., Leister-Tebbe H., Conte U., Raber S., Swanson R., Yan J.L., Aram J.A., Queiroz-Telles F. (2020). Safety, Efficacy and Pharmacokinetics of Anidulafungin in Patients 1 Month to <2 Years of Age with Invasive Candidiasis, Including Candidemia. Pediatr. Infect. Dis. J..

[108] Roilides E., Carlesse F., Leister-Tebbe H., Conte U., Yan J.L., Liu P., Tawadrous M., Aram J.A., Queiroz-Telles F. (2019). Anidulafungin A8851008 Pediatric Study Group. A Prospective, Open-label Study to Assess the Safety, Tolerability and Efficacy of Anidulafungin in the Treatment of Invasive Candidiasis in Children 2 to <18 Years of Age. Pediatr. Infect. Dis. J..

[109] Kriegel C., Festag M., Kishore R.S.K., Roethlisberger D., Schmitt G. (2019). Pediatric Safety of Polysorbates in Drug Formulations. Children.

[110] Saito J., Nadatani N., Setoguchi M., Nakao M., Kimura H., Sameshima M., Kobayashi K., Matsumoto H., Yoshikawa N., Yokoyama T. (2021). Potentially harmful excipients in neonatal medications: A multicenter nationwide observational study in Japan. J. Pharm. Health Care Sci..

[111] Sokou R., Palioura A.E., Kopanou Taliaka P., Konstantinidi A., Tsantes A.G., Piovani D., Tsante K.A., Gounari E.A., Iliodromiti Z., Boutsikou T. (2024). *Candida auris* Infection, a Rapidly Emerging Threat in the Neonatal Intensive Care Units: A Systematic Review. J. Clin. Med..

[112] Gamal A., Long L., Herrada J., Aram J., McCormick T.S., Ghannoum M.A. (2021). Efficacy of Voriconazole, Isavuconazole, Fluconazole, and Anidulafungin in the Treatment of Emerging *Candida auris* Using an Immunocompromised Murine Model of Disseminated Candidiasis. Antimicrob. Agents Chemother..

[113] Center for Diseases Control and Prevention (CDC). https://www.cdc.gov/candida-auris/hcp/clinical-care/index.html.

[114] Ramya G.M., Balakrishnan U., Chandrasekaran A., Abiramalatha T., Amboiram P., Sekar U., UshaDevi R. (2021). *Candida auris*, an emerging pathogen—Challenge in the survival of microprimies. Indian. J. Med. Microbiol..

[115] Talapko J., Juzbašić M., Matijević T., Pustijanac E., Bekić S., Kotris I., Škrlec I. (2021). Candida albicans—The Virulence Factors and Clinical Manifestations of Infection. J. Fungi.

[116] Junqueira J.C., Mylonakis E. (2023). Editorial: *Candida* biofilms. Front. Microbiol..

[117] Wijaya M., Halleyantoro R., Kalumpiu J.F. (2023). Biofilm: The invisible culprit in catheter-induced candidemia. AIMS Microbiol..

[118] Hoyer A.R., Johnson C.J., Hoyer M.R., Kernien J.F., Nett J.E. (2018). Echinocandin Treatment of Candida albicans Biofilms Enhances Neutrophil Extracellular Trap Formation. Antimicrob. Agents Chemother..

[119] Malinovská Z., Čonková E., Váczi P. (2023). Biofilm Formation in Medically Important Candida Species. J. Fungi.

[120] Cavalheiro M., Teixeira M.C. (2018). *Candida* Biofilms: Threats, Challenges, and Promising Strategies. Front. Med..

[121] Imbert C., Rammaert B. (2018). What Could Be the Role of Antifungal Lock-Solutions? From Bench to Bedside. Pathogens.

[122] Katragkou A., Kruhlak M.J., Simitsopoulou M., Chatzimoschou A., Taparkou A., Cotten C.J., Paliogianni F., Diza-Mataftsi E., Tsantali C., Walsh T.J. (2010). Interactions between human phagocytes and Candida albicans biofilms alone and in combination with antifungal agents. J. Infect. Dis..

[123] Chen Y.N., Hsu J.F., Chu S.M., Lai M.Y., Lin C., Huang H.-R., Yang P.H., Chiang M.C., Tsai M.H. (2022). Clinical and Microbiological Characteristics of Neonates with Candidemia and Impacts of Therapeutic Strategies on the Outcomes. J. Fungi.

[124] Kovács R., Majoros L. (2022). Antifungal lock therapy: An eternal promise or an effective alternative therapeutic approach?. Lett. Appl. Microbiol..

[125] Buonsenso D., Salerno G., Sodero G., Mariani F., Pisapia L., Gelormini C., Di Nardo M., Valentini P., Scoppettuolo G., Biasucci D.G. (2022). Catheter salvage strategies in children with central venous catheter-related or -associated bloodstream infections: A systematic review and meta-analysis. J. Hosp. Infect..

[126] Ghannoum M., Roilides E., Katragkou A., Petraitis V., Walsh T.J. (2015). The Role of Echinocandins in Candida Biofilm-Related Vascular Catheter Infections: In Vitro and In Vivo Model Systems. Clin. Infect. Dis..

[127] Wagener J., Loiko V. (2017). Recent Insights into the Paradoxical Effect of Echinocandins. J. Fungi.

[128] Miceli M.H., Bernardo S.M., Lee S.A. (2009). In Vitro analysis of the occurrence of a paradoxical effect with different echinocandins and *Candida albicans* biofilms. Int. J. Antimicrob. Agents..

[129] Simitsopoulou M., Peshkova P., Tasina E., Katragkou A., Kyrpitzi D., Velegraki A., Walsh T.J., Roilides E. (2013). Species-specific and drug-specific differences in susceptibility of Candida biofilms to echinocandins: Characterization of less common bloodstream isolates. Antimicrob. Agents Chemother..

[130] Ku T.S.N., Bernardo S.M., Lee S.A. (2011). In Vitro assessment of the antifungal and paradoxical activity of different echinocandins against *Candida tropicalis* biofilms. J. Med. Microbiol..

[131] Prażyńska M., Gospodarek-Komkowska E. (2019). Paradoxical growth effect of caspofungin on *Candida* spp. sessile cells not only at high drug concentrations. J. Antibiot.

[132] Iosifidis E., Papachristou S., Roilides E. (2018). Advances in the Treatment of Mycoses in Pediatric Patients. J. Fungi.

[133] Fortmann I., Hartz A., Paul P., Pulzer F., Müller A., Böttger R., Proquitté H., Dawczynski K., Simon A., Rupp J. (2018). German Neonatal Network. Antifungal Treatment and Outcome in Very Low Birth Weight Infants: A Population-based Observational Study of the German Neonatal Network. Pediatr. Infect. Dis. J..

[134] Ferreras-Antolín L., Irwin A., Atra A., Dermirjian A., Drysdale S.B., Emonts M., McMaster P., Paulus S., Patel S., Kinsey S. (2019). Neonatal Antifungal Consumption Is Dominated by Prophylactic Use; Outcomes from the Pediatric Antifungal Stewardship: Optimizing Antifungal Prescription Study. Pediatr. Infect. Dis. J..

[135] Chu M., Lin J., Wang M., Liao Z., Cao C., Hu M., Ding Y., Liu Y., Yue S. (2023). Restrictive Use of Empirical Antibiotics Is Associated with Improved Short Term Outcomes in Very Low Birth Weight Infants: A Single Center, Retrospective Cohort Study from China. Antibiotics.

[136] Zini T., Miselli F., D’Esposito C., Fidanza L., Cuoghi Costantini R., Corso L., Mazzotti S., Rossi C., Spaggiari E., Rossi K. (2024). Sustaining the Continued Effectiveness of an Antimicrobial Stewardship Program in Preterm Infants. Trop. Med. Infect. Dis..

[137] De Rose D.U., Ronchetti M.P., Santisi A., Bernaschi P., Martini L., Porzio O., Dotta A., Auriti C. (2024). Stop in Time: How to Reduce Unnecessary Antibiotics in Newborns with Late-Onset Sepsis in Neonatal Intensive Care. Trop. Med. Infect. Dis..

[138] Astorga M.C., Piscitello K.J., Menda N., Ebert A.M., Ebert S.C., Porte M.A., Kling P.J. (2019). Antibiotic Stewardship in the Neonatal Intensive Care Unit: Effects of an Automatic 48-Hour Antibiotic Stop Order on Antibiotic Use. J. Pediatric Infect. Dis. Soc..

[139] Kourti M., Chorafa E., Roilides E., Iosifidis E. (2023). Antifungal Stewardship Programs in Children: Challenges and Opportunities. Pediatr. Infect. Dis. J..

[140] Lestner J.M., Versporten A., Doerholt K., Warris A., Roilides E., Sharland M., Bielicki J., Goossens H. (2015). ARPEC Project Group. Systemic antifungal prescribing in neonates and children: Outcomes from the Antibiotic Resistance and Prescribing in European Children (ARPEC) Study. Antimicrob. Agents Chemother..

[141] Albahar F., Alhamad H., Abu Assab M., Abu-Farha R., Alawi L., Khaleel S. (2023). The Impact of Antifungal Stewardship on Clinical and Performance Measures: A Global Systematic Review. Trop. Med. Infect. Dis..

